# Baseline characteristics of children in the Early Glasses Study

**DOI:** 10.1007/s00417-024-06621-8

**Published:** 2024-09-05

**Authors:** J. S. Steltman, M. Nordmann, D. Sanders, W. L. Asjes-Tydeman, T. Dehpoor, I. Tissen, R. van Ommen, C. Wiersma-Hartman, M. M. van Keulen, D. Bakker, S. E. Loudon, H. J. Simonsz

**Affiliations:** 1 Dept. of Ophthalmology, Erasmus University Medical Center, Dr. Molewaterplein 40, Ee-1667, 3015 GD Rotterdam, Netherlands; 2 Public Health Services Limburg-North, Venlo, Netherlands; 3 Public Health Services Municipality Utrecht, Utrecht, Netherlands; 4 Public Health Services Brabant-Southeast, Eindhoven, Netherlands; 5 Public Health Services Utrecht, Zeist, Netherlands

**Keywords:** Hypermetropia, Astigmatism, Anisometropia, Amblyopia, Accommodative estropia, Glasses

## Abstract

**Purpose:**

The relationship between refractive error at age 1 and the risk of developing amblyopia or accommodative esotropia, and the protection offered by early glasses, is unknown. These are determined in the Early Glasses Study, a prospective, population-based, longitudinal, randomized controlled study. We report baseline findings.

**Methods:**

Healthy children aged 12–18 months were recruited at Children’s Healthcare Centres (CHCs) and received an entry orthoptic examination followed by cycloplegic retinoscopy. Children with amblyopia, strabismus, ophthalmic disease or very high refractive error were excluded. Those exceeding the AAPOS 2003 Criteria (> + 3.5D spherical equivalent (SE), > 1.5D astigmatism, > 1.5D anisometropia) were randomized into wearing glasses or not, and are followed-up by research orthoptists. Other children are followed-up by regular vision screening at CHCs and visual acuity is measured in all children at age 4.

**Results:**

Parents of 865 children were called, 123 were excluded. Of 742 children enrolled, 601 underwent the entry orthoptic examination at age 14.5 ± 1.7 months. Mean SE was + 1.73 ± 1.18D, astigmatism -0.70 ± 0.44D, anisometropia 0.21D (IQR: 0–0.25). Of 62 (10.3%) children exceeding the Criteria, 52 were randomized into wearing glasses or not. Of 539 other children, 522 are followed up at CHCs. In total, 31 were excluded: 2 had strabismus and amblyopia, 7 strabismus, 2 amblyopia suspect, 1 strabismus suspect, 1 squinting during sinusitis, 4 excessive refractive error, 9 myopia, 2 ptosis, 1 oculomotor apraxia, 1 Duane syndrome, 1 congenital nystagmus.

**Conclusion:**

Prevalence of strabismus (10/601) was as expected, but prevalence of amblyopia (2/601) was low, suggesting that common amblyopia develops later than generally thought.

****Key messages**:**

***What is known***

- High refractive errors cause amblyopia, but no study has determined the exact relationship between the kind and size of refractive error at age 1 and the risk to develop amblyopia, and assessed the protective effect of glasses in a controlled, population-based, longitudinal study.

***What is new***

- At baseline, 601 children received a full orthoptic examination followed by retinoscopy in cycloplegia at the age of 14.5 ± 1.7 months; 10.3% had high refractive error exceeding spherical equivalent > + 3.5D, > 1.5D astigmatism, > 1D oblique astigmatism or > 1.5D anisometropia.
- The prevalence of amblyopia was lower (0.3%) than expected, suggesting that most amblyopia develops after the first year of life.
- The prevalence of anisometropia, associated with amblyopia in older children, was low (0.8%).

**Supplementary information:**

The online version contains supplementary material available at 10.1007/s00417-024-06621-8.

## Introduction

Amblyopia has a prevalence of 3–3.5% and is mostly caused by refractive errors, strabismus, or both [1]. Strabismus is mostly noted by parents [2]. Amblyopia caused by refractive errors is usually detected by measurement of visual acuity (VA) at age 4–5 years [1]. Robert Ingram first advocated screening on refractive errors in very young children, assuming that prescription of glasses at an early age could prevent development of amblyopia and accommodative esotropia [3]. Accordingly, in several countries photoscreening and photorefraction at an early age and prescription of early glasses, are being considered [4]. An obstacle with prescription of early glasses is that the relationship between the size and type of the refractive error and the increase in chance of developing accommodative esotropia and/or amblyopia is unknown. Several studies, although not always population-based, nor prospective, longitudinal, or with randomization, have tried to determine its relationship [3, 5–8]. Although several studies have been performed, the relationship between the size and type of the refractive error and the increased chance of developing accommodative esotropia and/or amblyopia has not been quantified. Instead, the American Association for Pediatric Ophthalmology and Strabismus (AAPOS) has defined thresholds for referral from screening to an ophthalmologist or orthoptist. These are generally called the AAPOS Criteria. They have been first formulated in 2003 [9]. Revisions of the thresholds were published in 2013 and 2021 [10].

The Early Glasses Study (EGS) aims to determine the relative risk of various type of refractive error at age 1 to develop accommodative esotropia and/or amblyopia and the relative protection offered by early glasses. In this report we describe baseline characteristics of all children recruited, enrolled, excluded and randomized in the EGS. As a threshold for randomization we used the AAPOS 2003 Criteria (the “Criteria” in the following).

## Materials and methods

### Recruitment of youth health care regions

Participating CHCs were recruited by coordination of youth healthcare doctors, who were informed about the EGS by the research team. In the Netherlands the Youth Health Care (YHC) is responsible for monitoring and promoting the health of newborn children up to 18 years. The YHC is situated in approximately 880 Children’s Healthcare Centres (CHCs) under the responsibility of Municipal Health Services in 257 municipals, healthcare organizations (10), foundations (21), municipals (3), or a combination of above (51) [11].

### Recruitment and participant eligibility criteria

Children were recruited after visiting participating CHCs in the Netherlands, as part of screening for general health disorders and vaccinations. Parents of children aged 7 and 14 months received a brochure with information about the EGS. The brochure was available in different languages: Arabic, Dutch, English, German, Polish, and Turkish. Staff members of CHCs were not permitted by their employer to recruit, so recruitment consisted of scanning a QR-code from stickers, brochures or banners in participating CHCs, linked to a web-based registration-form.

After recruitment, parents were contacted by telephone by the research team for additional information and oral check of in- and exclusion criteria. Included were: children who could be examined between age ≥ 12 and < 18 months, born after 36 weeks gestation, registered at one of the participating CHCs, with voluntary consent for participation by both parents or legal representative or guardian and their willingness of complying with the study procedures. Excluded were: children who were not able to undergo the Entry Orthoptic Examination between age 12–18 months, children born prematurely or with perinatal birth damage, congenital syndromes, psychomotor retardation, known hereditary defects, cardiac disease, neurological disease, severe comorbidity, known ophthalmic diseases. Children who met the inclusion criteria and did not meet exclusion criteria were enrolled, and an appointment for the Entry Orthoptic Examination was made by the research orthoptist. It could happen that parents declined participation when an appointment would be made. Additional exclusion criteria could apply after the Entry Orthoptic Examination, as described below.

### Entry orthoptic examination

All parents of enrolled children were required to provide written consent by both parents or legal representative or guardian prior to the Entry Orthoptic Examination. The Entry Orthoptic Examination was performed by research orthoptists. Date of birth, country of birth of participating children as well as from their parents, place of residence, language class of the parents (Class 1: does not speak Dutch or English; Class 2: can make themselves poorly understood; Class 3: sufficiently understood; Class 4: accent; Class 5: fluent) and their self-reported level of education, according to the International Standard Classification of Education (ISCED; Low: ISCED 0–2, medium: ISCED 3–4, high: ISCED 5–8; Supplement 1) were recorded. For socioeconomic status (SES), mean SES of citizens in the cities and regions with participating CHCs was determined.

At the Entry Orthoptic Examination family history of amblyopia, strabismic amblyopia, strabismus, ptosis, cataract or other ophthalmic diseases were recorded. A full orthoptic examination was performed, including inspection, quality of eye pursuit, motility and pupillary reflex, corneal reflex and cover test to detect strabismus, and the 15-prism-diopter prism test for ocular dominance. Retinoscopy was performed at least 30 min after installing a drop of cyclopentolate hydrochloride 1% twice in each eye with at least 10 min in between. Finally fundoscopy was performed for assessment of the posterior retina and the optic nerve of the eye.

When amblyopia, strabismus, ptosis, cataract, nystagmus or other ophthalmic diseases were found, children were excluded after the Entry Orthoptic Examination and referred to an ophthalmologist and/or orthoptist (the “treating orthoptist” in the following). In case no preference of fixation was found, no dominance was found with the 15-prism-diopter prism test and monocular pursuit movements were adequate and symmetric, amblyopia was not considered to be present [12]. Children with excessive refractive error, higher than the Criteria twofold (spherical equivalent (SE) of >  + 7D, > 3D astigmatism, > 2D oblique astigmatism (10°-80°), and > 3D anisometropia), were excluded for randomization and referred as well, as it was considered unethical to leave these children without glasses. All findings of the Entry Orthoptic Examination were saved in Castor version 2023.1.1.1 (Castor Electronic Data Capture) and were reported to the CHCs. In addition, parents received a copy of the findings to hand over to the general practitioner of their child.

### Randomization

Examined children with a refraction exceeding the Criteria (SE of >  + 3.5D, > 1.5D astigmatism, > 1D oblique astigmatism or > 1.5D anisometropia), approximately 10%, were randomized in a 1:1 allocation ratio using block randomization to be fitted in glasses or not. Castor was used to perform the randomization procedure. Since signs of wearing glasses would be noticed by the research orthoptists, blinding was not feasible. The glasses were provided for free to the children assigned to the group with glasses.

### Follow-up (Orthoptic) examination

All randomized children will receive Follow-up Orthoptic Examinations performed by the research orthoptist, biannually to age 45–48 months. If a child reaches a primary endpoint by developing amblyopia or strabismus or developing another ophthalmic disease, the child will be referred to the treating orthoptist for treatment. In children randomized into wearing glasses, glasses wearing will be monitored electronically during at least 1 week from age 2 years. Examined children who did not exceed the Criteria, approximately 90%, are followed-up by youth healthcare doctors and -nurses at CHCs as part of general screening to age 45–48 months. At that age VA is first measured in all children in the Netherlands and these data will be communicated to the study center while children with unclear outcome will be reexamined by research orthoptists.

### Final orthoptic examination

All randomized children will receive a Final Orthoptic Examination and VA will be measured by the research orthoptist in presence of at least one other member of the research team, between age 45–48 months. Those who have reached a primary endpoint earlier during a Follow-up Orthoptic Examination and have been referred to the treating orthoptist will undergo a Final Orthoptic Examination and measurement of VA, between age 45–48 months, performed by the treating orthoptist, also attended by a member of the research team.

### Final VA measurement

Children who did not exceed the Criteria will be followed up to age 45–48 months at CHCs as part of general screening. They receive a Final VA Measurement, performed by youth healthcare doctors or -nurses. This data will be communicated to the study center. In case the result of the Final VA Measurement is unclear or unsatisfactory, the youth healthcare doctors will be asked to clarify or the child will be reexamined. In case children have been referred to an ophthalmologist and/or orthoptist during follow-up at the CHC, the results of orthoptic examinations will be collected from the treating orthoptist.

### Primary endpoints

The primary endpoints are development of accommodative esotropia or amblyopia before the age of 48 months. The VA measured from the worst eye will be used as a proxy for the degree of amblyopia.

### Supplemental procedure for establishing the diagnosis of amblyopia and strabismus

A deficiency in the study protocol was uncovered when assessing the application of the exclusion criteria amblyopia and strabismus. The study protocol had not been foreseen that research- and treating orthoptist would not be in agreement about the presence of amblyopia or strabismus. On September 5th, 2023, a supplemental procedure for establishing the diagnosis of amblyopia or strabismus was decided upon, after plenary discussion with the EGS Study Group:

1. The diagnosis of amblyopia or strabismus should be founded on the judgement of all research- and treating orthoptists who have examined the child.
2. These judgements are subsequently presented to the EGS Study Group including research- and treating orthoptists and a plenary decision of diagnosis will be reached (Supplement 2).

### Statistics and mathematical

Baseline characteristics are presented as means of continuous variables and as counts (percentages) of categorical variables. Characteristics of recruited, enrolled, excluded, and randomized children are shown in tables and figures. Significance values were determined using a two-tailed t-test analysis, with a significance value of p < 0.05 used for all analyses. All analyses were performed using IBM SPSS Statistics, version 28.0.1.0.

### Ethical approval

The study was approved by the Medical Ethics Review Committee (MERC) of the Erasmus University Medical Center on April 30th, 2021, and was carried out in accordance with the ethical conduct and juridical laws laid down in the Declaration of Helsinki, version 2013. First Entry Orthoptic Examination and randomization were on May 11th, 2021, and May 17th, 2021, respectively. Last child undergoing the Entry Orthoptic Examination and the last randomization took place on March 29th, 2023. The projected follow-up is approximately 3 years after the last Entry Orthoptic Examination. The study is registered at ClinicalTrials.gov, identifier: NCT04740593.

## Results

### Participating regions

After inviting YHC regions for participation in the EGS, CHCs covered by the YHC Limburg-North (covering the region North Limburg), YHC Utrecht (covering the city Amersfoort, Veenendaal, and three parts of the city of Utrecht: Kanaleneiland, Leidsche Rijn, Overvecht), YHC Brabant-Southeast (covering the city Eindhoven), and YHC North- and East-Gelderland (covering the city Harderwijk), decided to participate.

### Recruited children

In total, 888 parents showed their interest by scanning the QR-code and filling out the web-based registration-form. Twenty-three (2.6%) of these parents could not be reached by a recruiting telephone call. The remaining 865 parents received a telephone call by the research team for additional information, consisting of explanation of the aim of the study, background information of amblyopia and strabismus, explanation of the Entry Orthoptic Examination, possible side-effects of administered eye drops, randomization and follow-up procedure, and inclusion and exclusion criteria were checked.

**Fig. 1:**
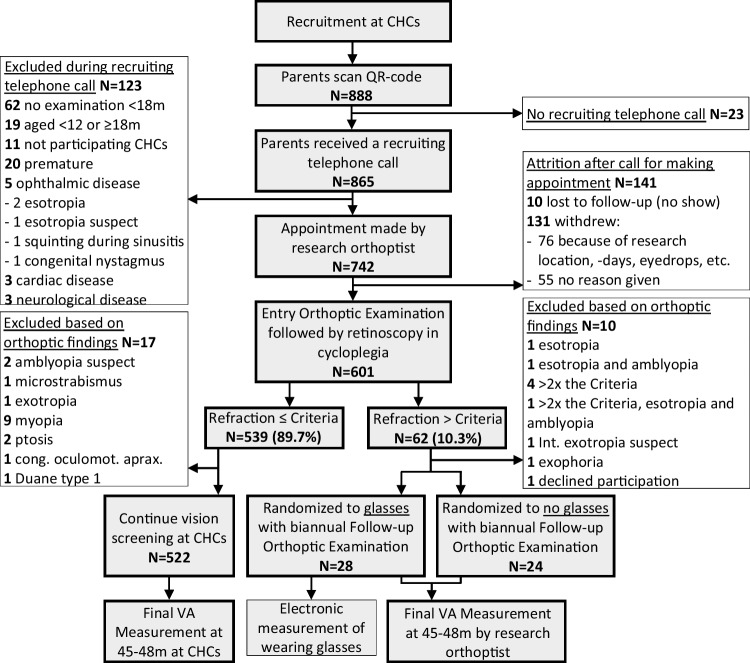
Flowchart from recruitment to Entry Orthoptic Examination and formation of three study groups. Children exceeding the Criteria (spherical equivalent >  + 3.5D, > 1.5D astigmatism, > 1D oblique astigmatism or > 1.5D anisometropia) were randomized into two groups, wearing glasses or not. Children not exceeding the Criteria continued vision screening as part of general screening at CHCs. All will have VA measured at 45 months using the same VA cards. Exclusion at each successive step is listed in white boxes on either side

In total, 123 (14.2%) children of contacted parents were excluded during the recruiting telephone call. Reasons for exclusion were prematurity (20 children), cardiac diseases (3), neurological diseases (3), previous treatment by an ophthalmologist and/or orthoptist (5, 2 of whom had esotropia, 1 was suspect for esotropia (later denied by treating orthoptist), 1 squinting during sinusitis, and 1 had congenital nystagmus). Other reasons for exclusion were: no possibility to schedule an appointment before age 18 months (62), < 12 or ≥ 18 months old during the entire period that children could receive an Entry Orthoptic Examination (19), or not registered at one of the participating CHCs (11) (Fig. 1). (Supplement 3).

### Enrolled children

After exclusion of 123 children during the recruiting telephone call and 23 parents of children who could not be reached, 742 children were eligible for an Entry Orthoptic Examination. However, 131 (15.1%) of these withdrew before the Entry Orthoptic Examination, 76 (8.8%) of whom declined because of one or more of the following: because of the distance to the research location (8), undesirable side-effects of cyclopentolate eyedrops (9), rejection by one of the parents (10), appointment could not be made (25), would impact child and family too much (26); 55 (6.4%) gave no reason. Ten (1.2%) additional children and their parents did not show up at the Entry Orthoptic Examination, and were considered to be lost to follow-up (Fig. 1) (Supplement 3).

### Examined children

Of all recruited and contacted, 601 received the Entry Orthoptic Examination. Of all examined, 230 children were recruited in CHCs in the region North Limburg (4.71% of 4886 children born in the municipalities with participating CHCs in this region in the recruiting period). Of the three parts of the city of Utrecht, 142 were recruited in CHCs in Leidsche Rijn, 9 in Kanaleneiland and 9 in Overvecht (3.72% of 4304 born). Of the remaining, 119 were recruited in CHCs in Eindhoven (2.89% of 4119 born); 50 in Harderwijk (3.9% of 1282 born); 23 in Veenendaal (4% of 575 born); 19 in Amersfoort (1.13% of 1681 born).

Parents of examined children were born in the Netherlands in 85.3%, as compared to 99.3% of the children. Highest language Class of parents was for most, 545 (90.7%), Class 5, 37 (6.2%) Class 4, 15 (2.5%) Class 3, none Class 2 or Class 1, of 4 language Class was unknown. (Supplement 4.1, 4.3, 4.4) Parents declared to have a low level of education in 1.7% of cases (13% in the general population aged 25–44 years) [13]. (Supplement 4.1 and 4.2).

Mean age at the Entry Orthoptic Examination was 14.5 ± 1.7 months, 52.6% were girl (*N* = 316). A positive first-degree family history for amblyopia was found in 116 (19.3%), strabismus 15 (2.5%), strabismus and amblyopia 55 (9.2%), ptosis 3 (0.5%), cataract 1 (0.2%). Seventy-six (12.6%) had a positive family history for other ophthalmic diseases, mostly high refractive errors or myopia (Supplement 4.3, 4.4).

Of 601 examined children, 62 (10.3%) had a refraction exceeding the Criteria, 52 (8.7%) were randomized into glasses (*N* = 28) or not (*N* = 24). Both groups will receive Follow-up Orthoptic Examinations biannually up to age 45–48 months. Nine (1.5%) were excluded because of: esotropia (1 of whom only had esotropia, the other of whom had manifest esotropia, +4.75D and +4.25D hypermetropia and preference of fixation whereas upon the diagnosis of amblyopia was made at the age of 14 months), intermittent exotropia (1), exophoria (1), or a refraction exceeding the Criteria twofold (5, 1 of whom had manifest esotropia and amblyopia with +8.25D and +6.75D hypermetropia at the age of 13 months) (Fig. 1) (Supplement 3). Parents of 1 child requested immediate referral because the refraction of their child exceeded the Criteria and an older participating sister, randomized into wearing no glasses, had developed strabismus (Supplement 3).

Of 539 (89.7%) with a refraction not exceeding the Criteria, 522 are followed-up at CHCs as part of general screening. Seventeen (2.8%) of these were excluded because of: amblyopia suspect (2, later denied by treating orthoptist), microstrabismus (1), exotropia (1), myopia (9), ptosis (2), congenital oculomotor apraxia (1) and Duane type 1 (1) (Fig. 1) (Supplement 3).

### Type, magnitude and distribution of all examined children (*n* = 601)

**Fig. 2:**
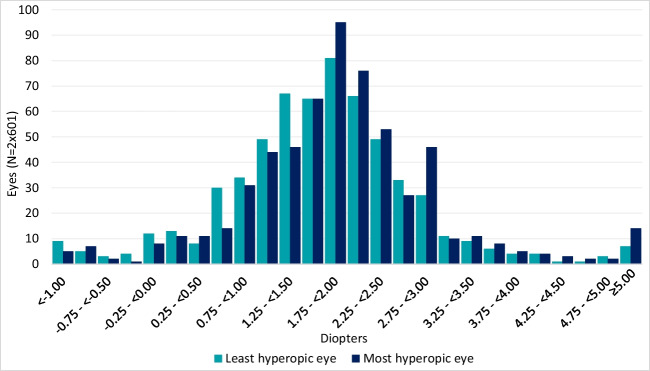
Distribution of spherical equivalent (SE) at 14.5 months. Mean SE of the most hypermetropic eye was + 1.84D (-2.50D to + 9.25D SD1.22), for the least + 1.63D (-3.00D to + 8.50D SD1.12)

**Fig. 3:**
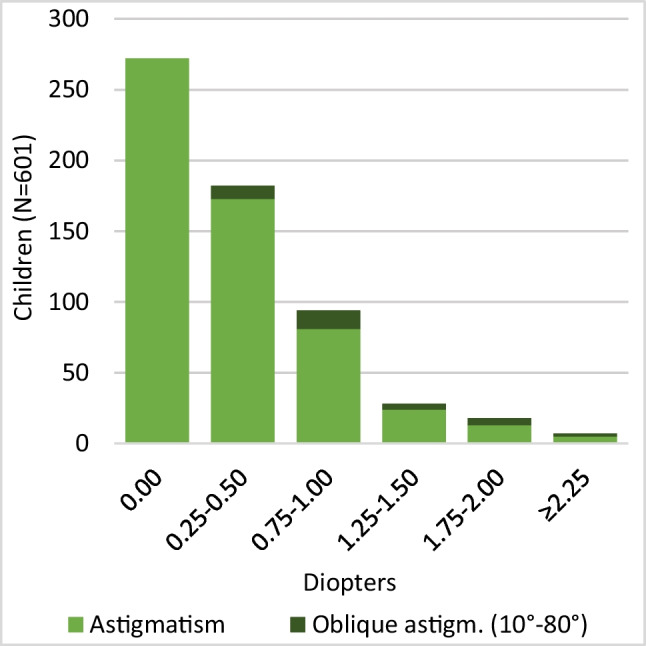
Distribution of astigmatism at 14.5 months

**Fig. 4:**
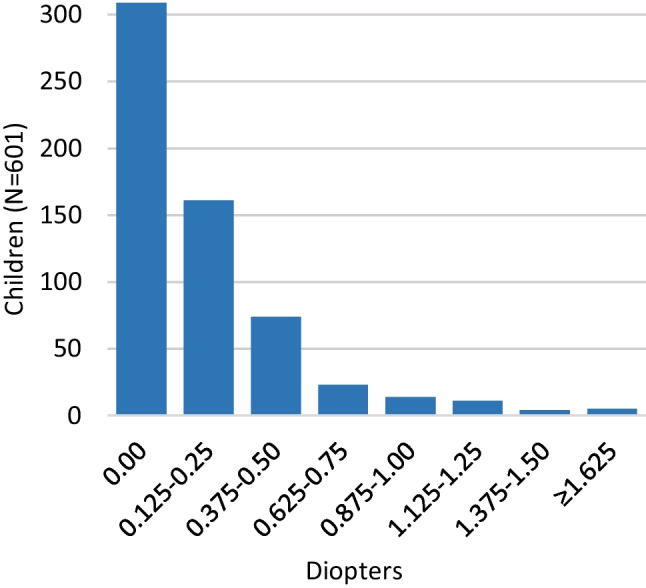
Distribution of anisometropia at 14.5 months. At the age of 14.5 months anisometropia is rare

Of 601 examined children, mean SE of 1202 eyes was + 1.73D (-3.00D to + 9.25D SD1.18) (Fig. 2). Mean SE of the most hypermetropic eye was + 1.84D (-2.50D to + 9.25D SD1.22) and for the least + 1.63D (-3.00D to + 8.50D SD1.12). In total, 329 (54.7%) children had astigmatism, with a mean of -0.70D (-3.50D to -0.25D SD0.44) (Fig. 3), mean oblique astigmatism was -0.94 (-2.75D to -0.25D SD0.59) (Fig. 3). Only few children had oblique astigmatism at age 14.5 months. Over half of examined children had no anisometropia, whereas 292 (48.6%) had anisometropia, with a mean of 0.21D. Five children had anisometropia between 1.625-2.625D (Fig. 4).

A positive first-degree family history for strabismic amblyopia was significantly more found in children with a refraction exceeding the Criteria, as compared to those with a refraction not exceeding the Criteria (Table 1). The prevalence of amblyopia and/or strabismus in first degree family members of children exceeding the Criteria is 46.8%. In first degree family members of children not exceeding the Criteria it is 29.3%. However, it must be realized that recruited children were estimated to have 3.5 first-degree family members. So the true prevalence will be much lower, but the difference is conspicuous however.

**Table 1:** Baseline findings of all 601 children who underwent the Entry Orthoptic Examination

	Refraction ≤ criteria (%)	Refraction > criteria (%)	*p*-value
Number	539	62	
Age at Entry Orthoptic Examination			
Mean ± SD (months)	14.5 ± 1.7	14.1 ± 1.5	p = 0.047
Girl	294 (54.5)	22(35.5)	p = 0.004
Family history (1st degree)			
Amblyopia and/or strabismus	158 (29.3)	29(46.8)	p = 0.005
Ptosis	3 (0.6)	0	p = 0.556
Cataract	1 (0.2)	0	p = 0.734
Other ophthalmic diseases	68 (12.6)	8(12.9)	p = 0.949

### Type and distribution of refractive error found in children whose refraction exceeded the Criteria (*N* = 62)

**Fig. 5:**
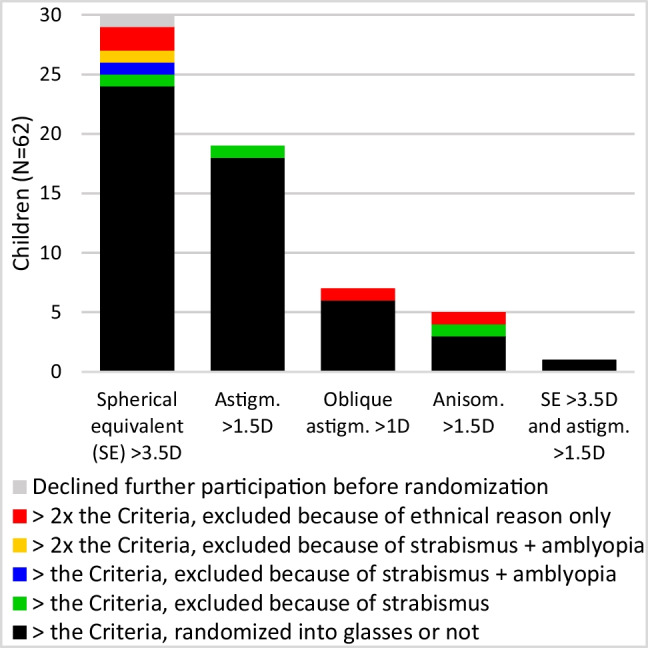
Distribution of refractive errors found in children whose refraction exceeded the Criteria. Children with strabismus, amblyopia or very high refractive error were excluded (color)

Of 62 with a refraction exceeding the Criteria, most had a SE > 3.5D (30, 48.4%), followed by > 1.5D astigmatism (19, 30.6%), only few had > 1.5D anisometropia (5, 8.1%) at age 14.5 months. Most of the children excluded because of strabismus (seven cases) or amblyopia (two cases),  had SE > 3.5D (Fig. 5).

In 2 of total 9 excluded children who exceeded the Criteria, and in 2 of total 17 excluded children who did not exceed the Criteria, one research orthoptist had not finished the complete Entry Orthoptic Examination before excluding the child, due to misunderstanding. The missing measurements of refractions were requested and received from the treating orthoptist.

## Discussion

The primary aims of the EGS are to determine the relative risk of various types of refractive error at age 1 to develop accommodative esotropia and/or amblyopia and, secondly, to determine the relative protection offered by early prescription of glasses. In this article, the demographics and baseline characteristics of recruited, enrolled, excluded and randomized children are reported.

Eight hundred sixty-five parents were contacted, 601 children underwent the Entry Orthoptic Examination at age 14.5 ± 1.7 months. We attempted to take a sample of the general population by recruiting at CHCs, which cover 97% of the children screening appointments in the Netherlands [14]. Nevertheless, an underrepresentation of low educated parents, 1.7%, as compared to 13% in the general population between age 25 and 44 years, was found. In an attempt to offset this bias, CHCs in Kanaleneiland and Overvecht, two regions in het city Utrecht with a lower SES and lower level of education were asked to participate (Supplement 4.2). Even there parents who signed up for participation declared to have a high education.

During the recruiting telephone call, 2 children were being treated for esotropia. At the Entry Orthoptic Examination, 2 had esotropia and amblyopia (both with a refraction exceeding the Criteria), 1 had esotropia, 1 microstrabismus, 1 Duane type 1, 1 suspect for intermittent exotropia, 1 exophoria, and 1 exotropia. In case no preference of fixation was found (no dominance was found with the 15-prism-diopters prism test and monocular pursuit movements were adequate and symmetric), amblyopia was not considered to be present. The judgement of an experienced orthoptist about the presence of amblyopia at age 1 is quite reliable [12]. The prevalence of amblyopia was lower than expected (*N* = 2; 0.3%), suggesting that amblyopia not caused by deprivation develops later than generally thought. In the previous birth-cohort Optimization of Amblyopia Screening (OVAS) study (*N* = 11,800) also a low prevalence of amblyopia was found (0.2%) in children examined at CHCs, at age 14 months [15]. One reason that contributes to the low prevalence of amblyopia found at the age of 14 months is that the spatial resolution of vision at that age is low and amblyopia affects high spatial resolution in the central visual field. In other words, the amblyopia may becomes manifest when the spatial resolution of the better eye improves naturally (personal communication Frank Schaeffel, August 15th, 2024). Also the prevalence of anisometropia exceeding 1.5D was low (*N* = 5; 0.8%).

The EGS will determine the relationship between refractive error at age 1 and the risk of developing amblyopia or accommodative esotropia, and the protection offered by early prescription of glasses, when VA is measured at age 45–48 months. Both at the Final Orthoptic Examination for randomized children (*N* = 52) and at the Final VA Measurement at CHCs for children who did not exceed the Criteria (*N* = 522), at age 45–48 months, the severity of amblyopia will be stratified according to their VA. It is uncertain whether the 52 children randomized into wearing glasses or not will yield sufficient statistical power to answer the second outcome measure: “What is the relative protection offered by early prescription of glasses?”. However, it will be possible to answer the first primary outcome: “What is the relative risk of various type of refractive error at age 1 to develop accommodative esotropia and/or amblyopia?”, since VA will be measured in all 601 children, at age 45–48 months.

## Supplementary information

Below is the link to the electronic supplementary material.

Supplementary file1 (DOCX 81.9 KB)

## Data Availability

The authors can be contacted for further queries on the data that support the findings of this study.
