# Supplementary material for: Baseline characteristics of children in the Early Glasses Study
Source: Graefes Arch Clin Exp Ophthalmol. 2024 Sep 5;263(2):555–63. doi: 10.1007/s00417-024-06621-8 (PMC11868130; doi:10.1007/s00417-024-06621-8)
Supplement: Supplementary file 1 — Supplementary file1 (DOCX 81.9 KB) [file 417_2024_6621_MOESM1_ESM.docx]

**Supplemental material**

***Supplement 1 Specification International Standard Classification of Education (ISDEC)***

Level of education, low, medium, high, was stratified according to the International Standard Classification of Education (ISCED; Low: ISCED 0-2, medium: ISCED 3-4, high: ISCED 5-8) [1]:

**Supplement table 1** Specification International Standard Classification of Education (ISCED)

| **Level of education** | **ISCED level** | **Description** |
| --- | --- | --- |
| Low | ISCED 0 | Early childhood education |
|  | ISCED 1 | Primary education |
|  | ISCED 2 | Lower secondary education |
| Medium | ISCED 3 | Upper secondary education |
|  | ISCED 4 | Post-secondary non-tertiary education |
| High | ISCED 5 | Short-cycle tertiary education |
|  | ISCED 6 | Bachelor’s or equivalent level |
|  | ISCED 7 | Master’s or equivalent level |
|  | ISCED 8 | Doctoral or equivalent level |

***Supplement 2 Supplemental procedure for establishing the diagnosis in children excluded at the Entry Orthoptic Examination or when children are referred at a Follow-up Orthoptic Examination***

A deficiency in the study protocol was uncovered when assessing the application of the exclusion criteria amblyopia and strabismus. The study protocol had not been foreseen that research- and treating orthoptist would not be in agreement about the presence of amblyopia or strabismus. On September 5th, 2023, a supplemental procedure for establishing the diagnosis of amblyopia or strabismus was decided upon, after plenary discussion with the EGS Study Group. The findings at orthoptic examination of excluded and referred children, as judged by the treating orthoptist, have been requested. According to the supplemental procedure, the judgement of research- and treating orthoptist are presented to the EGS Study Group and a plenary decision of diagnosis is reached. After discussing all excluded children, reason for exclusion of 7 children were subject of discussion since research- and treating orthoptist disagreed about the diagnosis, or reason for exclusion was misreported. (Supplement table 2)

Reason for exclusion of child 1 was initially misreported as “esotropia combined with >2x the Criteria”, existing amblyopia was not reported due to misinterpretation of data-analysis. Child 2 had probably developed amblyopia between age 13.1 and 18.4 months, as agreed in EGS Study Group meeting. Final diagnosis of child 3 was “esotropia in combination with amblyopia”. During the cover test at the Entry Orthoptic Examination, fixation of OD could be maintained for a short moment, therefore, development of amblyopia seems plausible. Child 4 was reassessment by the principal investigator, who found “esophoria and exceeding the Criteria”, therefore the child was randomized instead of excluded. Due to database limitations a second record was created for randomization, which was not clear documented. Therefore the child was previous misreported as excluded as well as randomized. Final diagnosis of child 5 was “suspect for intermittent exotropia”. Definitive diagnosis will be determined at age 45 months, since if exotropia exist, it will become more apparent in older age. Final diagnosis of child 6 was “no amblyopia”. During the Entry Orthoptic Examination the child was amblyopia suspect, due to a saccadic eye movement OS. However, during follow-up by the treating orthoptist, no signs of amblyopia were found. Final diagnosis of child 7 was “no amblyopia”, since referral was based on anisometropia of 1.25D and a positive family history for strabismus and amblyopia. According to the study protocol, child 6 and 7 should not have been referred, since an amblyopia could have been developed later during follow-up. Therefore, we will provide an as treated analysis as well when all data of Final Orthoptic Examination is collected.

**Supplement table 2** Overview excluded children of whom two research orthoptist or research orthoptist and treating orthoptist were not in agreement about the presence of amblyopia or strabismus or were previously misreported

*G+: glasses, G-: no glasses, P+: patching, P-: no patching*

| **Nr.** | **Age** | **Refraction OD** | **Refraction OS** | **Reason for exclusion research orthoptist** | **Findings treating orthoptist or after re-examination principal investigator*** | | |
| --- | --- | --- | --- | --- | --- | --- | --- |
| 1 | 13 | S+8.25D | S+6.75D | Amblyopia, esotropia, >2x the Criteria | Amblyopia, esotropia | G+\|P- | (13) |
| 2 | 13.1 | S+4.75D | S+6.75D | Esotropia | Amblyopia, esotropia | G+\|P- | (18.4) |
| 3 | 13.4 | S+4.50D | S+4.25D=C-0.50x125 | Esotropia | Amblyopia, esotropia | G-\|P+ | (13.6) |
| 4 | 13.5 | S+6.00D=C-1.25x180 | S+4.00D | Esotropia | No esotropia* |  | (15.5) |
| 5 | 14 | S+2.00D=C-2.75x90 | S+2.25D=C-1.50x90 | Int. exotropia | No exotropia | G-\|P- | (15.2) |
| 6 | 14.2 | S+1.25D=C-0.50x35 | S+1.25D=C-1.00x145 | Amblyopia suspected | No amblyopia | G-\|P- | (15.2) |
| 7 | 15.4 | S+1.75D | S+0.50D | Amblyopia suspected | No amblyopia | G-\|P- | (18.3) |

***Supplement 3***

Below, an overview is given of excluded children during recruiting telephone call, children whose refraction exceeded the Criteria and were excluded because of orthoptic findings and children whose refraction not exceeded the Criteria and were excluded because of orthoptic findings. Children lost in follow-up, declined participation or did not show up are not noted.

*Legend: G+: glasses, G-: no glasses, P+: patching, P-: no patching, *children who were subject of discussion after adjustment data processing policy*

| **Amount children** | **Age (mos.)** | **Refraction OD** | **Refraction OS** | **Reason for exclusion or declining participation** | **Findings treating orthoptist (age in mos.)** | | |
| --- | --- | --- | --- | --- | --- | --- | --- |
| ***Recruited and excluded during recruiting telephone call (N=123)*** | | | | | | | |
| 2 | - | - | - | Born after 31 weeks gestation | - |  |  |
| 3 | - | - | - | Born after 32 weeks gestation | - |  |  |
| 2 | - | - | - | Born after 33 weeks gestation | - |  |  |
| 2 | - | - | - | Born after 34 weeks gestation | - |  |  |
| 11 | - | - | - | Born after 35 weeks gestation | - |  |  |
| 2 | - | - | - | Cardiac disease: congenital heart defect | - |  |  |
| 1 | - | - | - | Cardiac disease: double aortic arch |  |  |  |
| 1 | - | - | - | Neurologic disease: cerebrovasculair accident, epilepsy | - |  |  |
| 1 | - | - | - | Neurological disease: absence seizure |  |  |  |
| 1 | - | - | - | Neurological disease: cerebral paresis |  |  |  |
| 2 | - | - | - | Ophthalmic disease: esotropia | - |  |  |
| 1 | - | - | - | Ophthalmic disease: suspect esotropia (later not confirmed) | - |  |  |
| 1 | - | - | - | Ophthalmic disease: anisometropia and minor eye movement disorder | - |  |  |
| 1 | - | - | - | Ophthalmic disease: congenital nystagmus | - |  |  |
| 62 | - | - | - | No possibility to schedule an appointment <18 mos. | - |  |  |
| 19 | - | - | - | Aged <12 or ≥18 months during examination period | - |  |  |
| 11 | - | - | - | Not registered at one of the participating CHCs | - |  |  |
| ***No recruiting telephone call (N=23)*** | | | | | | | |
| 23 | - | - | - | Could not be reached for a recruiting telephone call | - |  |  |
| ***Parents withdrew when study orthoptist called for making an appointment for the Entry Orthoptic Examination (N=131)*** | | | | | | | |
| 76 | - | - | - | Declined participation because of one or more reasons: distance to the research location, undesirable side-effects of cyclopentolate hydrochloride 1%, one parent did not approve participation, possible examination-days, too much impact on the child and family. | - |  |  |
| 55 | - | - | - | Did not want to give a reason for declining participation | - |  |  |
| ***Children lost to follow-up (N=10)*** | | | | | | | |
| 10 | - | - | - | Did not show up at the Entry Orthoptic Examination | - |  |  |
| ***Children > the Criteria excluded based on orthoptic findings (N=10)*** | | | | | | | |
| 1 | 12.4 | S+4.75D | S+7.75D=C-1.00 x 180 | >2x the Criteria | Probable amblyopia | G+\|P+ | (12.9) |
| 1 | 12.6 | S+0.75D=C-2.00 x 135 | S+0.75D=C-2.75 x 45 | >2x the Criteria | No amblyopia | G-\|P- | (14.7) |
| 1 | 12.7 | S+9.25D | S+8.75D=C-0.50 x 180 | >2x the Criteria | No amblyopia | G+\|P- | (17.1) |
| 1 | 15.2 | S+7.25D | S+7.25D | >2x the Criteria | No amblyopia | G-\|P- | (16.2) |
| 1* | 13 | S+8.25D | S+6.75D | Amblyopia, esotropia, >2x the Criteria | Amblyopia, esotropia | G+\|P- | (13) |
| 1* | 13.1 | S+4.75D | S+6.75D | Esotropia | Amblyopia, esotropia | G+\|P+ | (18.4) |
| 1* | 13.4 | S+4.50D | S+4.25D=C-0.50 x 125 | Esotropia | Amblyopia, esotropia | G-\|P+ | (13.6) |
| 1* | 14 | S+2.00D=C-2.75 x 90 | S+2.25D=C-1.50 x 90 | Int. exotropia | No exotropia | G-\|P- | (15.2) |
| 1 | 16.3 | S+6.00D=C-1.00 x 180 | S+7.00D=C-1.00 x 90 | Exophoria | Exophoria | G+\|P- | (17.1) |
| 1 | 14.7 | S+5.25D | S+5.75D=C-1.00 x 90 | Declined further participation because an older sibling already participating in the EGS was randomized and had developed strabismus during follow-up. | - |  |  |
| ***Children ≤ the Criteria excluded based on orthoptic findings (N=17)*** | | | | | | | |
| 1* | 14.2 | S+1.25D=C-0.50 x 35 | S+1.25D=C-1.00 x 145 | Amblyopia suspected | No amblyopia | G-\|P- | (15.2) |
| 1* | 15.4 | S+1.75D | S+0.50D | Amblyopia suspected | No amblyopia | G-\|P- | (18.3) |
| 1 | 16.6 | S+1.00D | S+1.00D | Microstrabismus | No amblyopia, esotropia | G-\|P- | (18.7) |
| 1 | 12.6 | S+0.50D=C-0.50 x 90 | S 0.00D | Exotropia | Exotropia | G-\|P- | (14.9) |
| 1 | 14.3 | S+1.25D | S+1.25D | Ptosis | No amblyopia | G-\|P- | (19.1) |
| 1 | 16.2 | S+2.00D | S+3.00D=C-1.00 x 180 | Ptosis | Probable amblyopia | G-\|P- | (18.9) |
| 1 | 14.7 | S-0.50D=C-3.50 x 180 | S-1.50D=C-1.50 x 180 | Myopia | Myopia | G-\|P- | (20.3) |
| 1 | 14 | S-0.25D=C-1.75 x 90 | S-0.25D=C-1.75 x 90 | Myopia | Myopia | G-\|P- | (14.8) |
| 1 | 15.2 | S0.00D=C-1.75 x 135 | S-1.00D=C-1.00 x 35 | Myopia | Myopia | G-\|P- | (19.5) |
| 1 | 12.3 | S-1.50D=C-0.50 x 90 | S-1.50D=C-0.50 x 80 | Myopia | Myopia | G-\|P- | (12.7) |
| 1 | 14.3 | S-1.75D=C-0.50 x 90 | S-0.75D=C-0.25 x 90 | Myopia | Myopia | G-\|P- | (15.8) |
| 1 | 14 | S-1.00D | S-1.00D | Myopia | Myopia | G-\|P- | (25) |
| 1 | 12.2 | S-1.00D | S-1.00D | Myopia | Myopia | G-\|P- | (12.5) |
| 1 | 12.8 | S-0.75D | S-1.00D | Myopia | Myopia | G-\|P- | (15.1) |
| 1 | 14.1 | S-3.00D | S-2.50D | Myopia | Myopia | G-\|P- | (17.5) |
| 1 | 12.2 | S+0.50D | S0.00D=C-1.00 x 0 | Con. oculomotor apraxia | Con. oculomotor apraxia | G-\|P- | (12.7) |
| 1 | 13.4 | Not measured | Not measured | Duane type 1 | No amblyopia, Duane type 1 | G-\|P- | (17.3) |

***Supplement 4.1***

Since language barriers affect the compliance of wearing occlusion patches for amblyopia treatment [2], we assumed that compliance of wearing glasses could be affected by language barriers as well. We expected language barriers to exist mostly when parents were not born in the Netherlands. Therefore, we distributed language level per country of birth. (Supplement table 3)

Parents declared a high level of education in 67.6% of cases (52% in the general population aged 25-44 years) [3]. In total, 72.4% of the mothers had declared to have an high level of education, 25.8% medium, 1.3% low and 0.5% was unknown. Fathers had declared to have an high level of education in 62.9%, 33.4% medium, 2% low and 1.7% was unknown. Remarkable is a high proportion of parents born in Poland who had declared to have a medium- or low level of education, of whom most were living in the region North Limburg, known to be a region with many foreign workers. Also remarkable was declaration of a high level of education in all parents born in India. Almost all of whom were living in the city Leidsche Rijn, known as a city with many expats [4]. The city Eindhoven has also many expats because of technology companies dispersed over the region, but no remarkable specific group of parents with the same country of birth could be extracted.

Declared low level of education is proportionally more found in mothers born in the Middle East and Africa, and fathers born in Middle-/South-America and Africa. However, this might be due to a small number of parents not being born in Europe.

**Supplement table 3** Distribution language level and level of education per country of birth

*Cl. = Class; Level of education, according to the International Standard Classification of Education (ISCED; Low: ISCED 0-2, medium: ISCED 3-4, high: ISCED 5-8 (Supplement table 1).*

| **Country of birth** | **Language level** | | | | | | **Level of education** | | | |
| --- | --- | --- | --- | --- | --- | --- | --- | --- | --- | --- |
|  | Cl. 5 | Cl. 4 | Cl. 3 | Cl. 2 | Cl. 1 | Unknown | High | Medium | Low | Unknown |
| Afghanistan (N=1) | - | - | - | - | - | 1 | 1 | - | - | - |
| Albania (N=2) | - | 2 | - | - | - | - | 2 | - | - | - |
| Argentina (N=2) | 1 | - | 1 | - | - | - | 2 | - | - | - |
| Belgium (N=1) | - | 1 | - | - | - | - | 1 | - | - | - |
| Bosnia and Herzegovina (N=2) | 1 | 1 | - | - | - | - | 1 | 1 | - | - |
| Brazil (N=9) | 5 | - | 3 | - | - | 1 | 7 | 2 | - | - |
| Caribbean Netherlands (N=3) | - | - | - | - | - | 3 | 2 | 1 | - | - |
| Colombia (N=5) | 5 | - | - | - | - | - | 4 | - | 1 | - |
| Czech Republic (N=2) | 1 | - | 1 | - | - | - | 1 | 1 | - | - |
| Denmark (N=3) | - | - | - | - | - | 3 | - | - | - | 3 |
| Egypt (N=2) | - | 1 | - | - | - | 1 | 2 | - | - | - |
| France (N=2) | - | 2 | - | - | - | - | 2 | - | - | - |
| French Guyana (N=1) | 1 | - | - | - | - | - | - | 1 | - | - |
| Germany (N=14) | 3 | 7 | - | - | - | 4 | 11 | 2 | 1 | - |
| Greece (N=4) | - | 1 | 2 | - | - | 1 | 4 | - | - | - |
| Hungary (N=1) | 1 | - | - | - | - | - | - | 1 | - | - |
| India (N=16) | 4 | 9 | - | - | - | 3 | 16 | - | - | - |
| Indonesia (N=3) | - | 2 | 1 | - | - | - | 3 | - | - | - |
| Iran (N=5) | - | 3 | - | - | - | 2 | 4 | 1 | - | - |
| Iraq (N=2) | - | - | - | - | - | 2 | 2 | - | - | - |
| Italy (N=2) | - | 2 | - | - | - | - | 2 | - | - | - |
| Lebanon (N=1) | - | - | 1 | - | - | - | 1 | - | - | - |
| Libya (N=1) | - | - | - | 1 | - | - | - | - | 1 | - |
| Lithuania (N=2) | - | 1 | - | - | - | 1 | 2 | - | - | - |
| Madagascar (N=1) | - | - | - | - | - | 1 | 1 | - | - | - |
| Marocco (N=11) | 7 | - | - | 1 | - | 3 | 7 | 3 | 1 | - |
| Mexico (N=4) | 1 | 3 | - | - | - | - | 4 | - | - | - |
| Nepal (N=2) | - | - | 1 | - | - | 1 | 2 | - | - | - |
| Netherlands (N=1019) | 619 | 1 | - | - | - | 399 | 675 | 329 | 11 | 4 |
| Nigeria (N=1) | - | - | - | - | - | 1 | 1 | - | - | - |
| North Macedonia (N=2) | - | - | 1 | - | - | 1 | 2 | - | - | - |
| Peru (N=2) | - | 1 | 1 | - | - | - | 1 | 1 | - | - |
| Philippines (N=2) | 2 | - | - | - | - | - | 2 | - | - | - |
| Poland (N=16) | - | 4 | 8 | - | - | 4 | 5 | 8 | 3 | - |
| Portugal (N=2) | 2 | - | - | - | - | - | 2 | - | - | - |
| Russia (N=1) | - | - | 1 | - | - | - | 1 | - | - | - |
| Singapore (N=1) | 1 | - | - | - | - | - | 1 | - | - | - |
| Slovenia (N=1) | - | - | - | - | - | 1 | 1 | - | - | - |
| Somalia (N=2) | - | 1 | - | - | - | 1 | - | 2 | - | - |
| Spain (N=10) | 3 | 3 | - | - | - | 4 | 9 | - | 1 | - |
| Sri Lanka (N=2) | 1 | - | - | - | - | 1 | 2 | - | - | - |
| Suriname (N=2) | 2 | - | - | - | - | - | 2 | - | - | - |
| Syria (N=7) | - | 4 | 2 | - | - | 1 | 5 | 1 | 1 | - |
| Turkey (N=1) | - | 1 | - | - | - | - | 1 | - | - | - |
| Ukraine (N=1) | - | - | 1 | - | - | - | - | 1 | - | - |
| United Kingdom (N=4) | 3 | - | - | - | - | 1 | 4 | - | - | - |
| United States (N=2) | 1 | 1 | - | - | - | - | 1 | 1 | - | - |
| Venezuela (N=3) | 1 | - | 1 | - | - | 1 | 3 | - | - | - |
| Vietnam (N=1) | - | 1 | - | - | - | - | 1 | - | - | - |
| South Africa (N=7) | 3 | 2 | - | - | - | 2 | 7 | - | - | - |
| South Korea (N=2) | - | 1 | - | - | - | 1 | 2 | - | - | - |
| Unknown (N=7) | - | - | - | - | - | 7 | 1 | - | - | 6 |

Comparing the declared level of education of parents of examined children within each region with participating CHCs, show in most regions an high amount of high level of education (Supplement Fig. 1, Supplement Fig. 2).

**Supplement Fig. 1** Distribution level of education mothers

**Supplement Fig. 2.** Distribution level of education fathers

***Supplement 4.2***

The declared level of education was not completely comparable with what we expected based on the SES. Of the regions with participating CHCs, the SES was below the mean (0 throughout the Netherlands) in Kanaleneiland and Overvecht, but ranged in all regions with participating CHCs from -0.418 till 0.446, in Overvecht and Amersfoort, respectively. Surprisingly, in Kanaleneiland (with a SES of -0.203) and Overvecht, many parents declared to have a high level of education as well. However, this could also be due to a low number of participating children in Kanaleneiland and Overvecht, both 9, or because of more interest in participation in scientific research when parents have a high level of education. (Supplement table 4).

**Supplement table 4** Distribution level of SES per region with participating CHCs in 2021 [5]

| **Region** | **<-0.2** | **-0.2 < -0.1** | **-0.1 < 0.0** | **0.0 < 0.1** | **0.1 < 0.2** | **> 0.2** |
| --- | --- | --- | --- | --- | --- | --- |
| Amersfoort |  |  |  |  |  | 0.446 |
| Eindhoven |  |  |  | 0.027 |  |  |
| Harderwijk |  |  |  |  | 0.125 |  |
| North Limburg |  |  |  |  | 0.111 |  |
| Kanaleneiland | -0.203 |  |  |  |  |  |
| Leidsche Rijn |  |  |  |  |  | 0.329 |
| Overvecht | -0.418 |  |  |  |  |  |
| Veenendaal |  |  |  |  | 0.127 |  |

***Supplement 4.3 Baseline findings examined and randomized***

All 52 randomized were born in Europe (100%), as well as most of their parents (81.7%). Highest language Class of parents of randomized children, was mostly Class 5 (94.2%), followed by Class 4 (5.8%). None had language Class 3, 2 or 1. In total, 63.5% of the parents had declared an high level of education, 33.7% medium, 1.9% low, and 1% was unknown. Mean age at the Entry Orthoptic Examination was 14.2±1.5 months, 35% of whom were girl (N=18). A positive first-degree family history was found in 13, 11 and 2, for amblyopia, strabismic amblyopia and strabismus, respectively. A positive family history for ptosis and cataract was not found. Eight had a positive first-degree family history with other ophthalmic diseases, of which most were high refractive errors or myopia.

***Supplement 4.4 Baseline findings examined and followed-up at CHCs***

Of 522 children, all were born in the Netherlands, except 1 child who was born in Spain and 1 in South-Korea, as well as most of their parents (85.7%). Highest language Class of most parents was Class 5 (90.8%), followed by Class 4 and Class 3, 6.3% and 2.5%, respectively. None had language Class 2 or 1. Of 0.4% language Class was unknown. In total, 68.4% of the parents had declared an high level of education, 29.1% medium, 1.7% low, and 0.8% was unknown. Mean age at the Entry Orthoptic Examination was 14.5±1.7 months, 54.6% of whom were girl (N=285). A positive first-degree family history for amblyopia, strabismic amblyopia, strabismus, ptosis, cataract was found in 97, 42, 13, 2, and 1 respectively. In total, 65 had a positive first-degree family history with other ophthalmic diseases, of which most were high refractive errors or myopia.

**References Supplemental material**

1. UNESCO Institute for Statistics (2012) International Standard Classification of Education ISCED 2011. https://uis.unesco.org/en/topic/international-standard-classification-education-isced. Accessed 28 March 2024.
2. Loudon SE, Fronius M, Looman CW et al (2006) Predictors and a remedy for noncompliance with amblyopia therapy in children measured with the occlusion dose monitor. Invest Ophthalmol Vis Sci 47:4393-4400. 10.1167/iovs.05-1428
3. Ministerie van Volksgezondheid, Welzijn en Sport (2023) Sociaaleconomische status | Opleiding. https://www.vzinfo.nl/sociaaleconomische-status/opleiding. Accessed 7 April 2024.
4. AlleCijfers.nl (2023) Statistieken wijk Leidsche Rijn. https://allecijfers.nl/wijk/wijk-09-leidsche-rijn-utrecht/. Accessed 25 January 2024.
5. Centraal Bureau voor de Statistiek (2023) Sociaal-economische status per postcode, 2020 en 2021. https://www.cbs.nl/nl-nl/maatwerk/2023/33/sociaal-economische-status-per-postcode-2020-en-2021. Accessed 25 January 2024.
